# Association of resistome abundance with hyperuricaemia in elderly individuals: a metagenomics study

**DOI:** 10.3389/frmbi.2024.1384703

**Published:** 2024-07-11

**Authors:** Zhiyang Liu, Yingbo Shen, Yulin Fu, Da Sun, Liang Li, Ziquan Lv

**Affiliations:** ^1^ School of Public Health, Hengyang Medical School, University of South China, Hengyang, China; ^2^ Central Laboratory Department, Shenzhen Center for Disease Control and Prevention, Shenzhen, China; ^3^ National Key Laboratory of Veterinary Public Health Safety, College of Veterinary Medicine, China Agricultural University, Beijing, China; ^4^ Department of Physiology, Hengyang Medical School, University of South China, Hengyang, China

**Keywords:** hyperuricaemia, microbiota, resistome, metagenomics, elderly people

## Abstract

**Introduction:**

Hyperuricaemia (HUA), one of chronic diseases, has an increased prevalence and is related to diseases such as gout, arthritis, infectious diseases, etc. Antimicrobial resistance (AMR) in the gut is considered as an atypical chronic disease, and poses risk to human health. The gut microbiome has been proved to be a reservoir for AMR and play an important role in HUA patients. The microbial characteristics of the gut in individuals with HUA have been previously explored, however, the characteristics of the resistome in individuals with HUA have remained largely unexplored.

**Methods:**

Thus, we investigated the landscape of the AMR in individuals with HUA and without HUA, and the potentially influential factors in a case-control study using metagenomics-based approaches.

**Results:**

We found that drinking juice and abnormal stool were risk factors associated with HUA. The taxonomic diversity of gut microbiota in individuals with HUA was lower than that in non-HUA individuals. Notably, a higher abundance and diversity of the resistome (entire antimicrobial resistance genes) was observed in individuals with HUA (median: 1.10 vs. 0.76, *P* = 0.039, U-test), especially in tetracycline resistance genes (median: 0.46 vs. 0.20, *P* < 0.001, U-test), which are associated with more complex mobile genetic elements (MGEs) in individuals with HUA. Furthermore, we found that a higher abundance of the resistome was positively correlated with uric acid (UA) levels and affected by several host-associated factors (mainly dietary habits). Specifically, pork consumption and the consumption of root and tuber vegetables were identified as contributing factors. We also found a higher abundance of virulence genes (VGs), mostly related to adherence, antimicrobial activity, competitive advantage, and exoenzymes, in the gut microbial community of individuals with HUA.

**Discussion:**

All findings revealed higher activity of the resistome and pathogenicity of the microbiota in individuals with HUA, indicating a higher health risk in the elderly HUA population.

## Highlights

- A higher abundance and diversity of resistome were observed in hyperuricaemic (HUA) individuals than in non-hyperuricaemic individuals.
- A positive correlation was identified between the resistome and uric acid (UA) levels, and host-associated factors were correlated with a higher abundance of the resistome in individuals with HUA.
- The microbiota of HUA individuals was associated with higher abundance of resistome and VGs than those in non-HUA individuals, which may pose a threat to human health.

## Introduction

Chronic diseases represent a pressing global health issue, with the percentage of deaths caused by them increasing from 67% to 74% over the past decade (Thomas et al., 2023). With progress of urbanisation and economic growth, people’s lifestyles and dietary patterns have greatly changed in last several decades; this has been accompanied by higher incidence of chronic diseases such as diabetes, hypertension, and hyperuricaemia (HUA) (Luo and Wang, 2022). Moreover, the mortality rates associated with these chronic diseases, particularly among elderly individuals (aged ≥ 60) have shown an increase from 2005 to 2020, indicating a heightened risk to elderly people (Cai et al., 2022a). Another study revealed that the proportion of deaths caused by chronic diseases among residents (aged ≥ 65) in China rose annually from 2004 (89.82%) to 2018 (91.41%) (Xia et al., 2021). In addition to deaths, chronic diseases impose an economic burden on society, especially in countries like India and China that have a large proportion of large elderly population (Xu and Yang, 2022). Furthermore, many countries are entering an ageing society stage, with China officially entering this stage (Fang et al., 2020). Hence, the attention on the elderly population with chronic diseases has escalated globally owing to increased mortality rates and economic burdens.

Numerous chronic diseases are linked to nutritional and metabolic disorders, especially those directly caused by malnutrition or metabolic dysregulation (Li et al., 2022). HUA, one of the chronic diseases resulting in arthritis and gout, has witnessed an increased prevalence in recent years worldwide (Yu and Cheng, 2020) as well as in Chinese adults (from 2015 to 2019, the prevalence rose from 11.1% to 14.0%.) (Liu et al., 2014; Zhang M. et al., 2021) and elderly (from 2010 to 2019, the prevalence maybe rose from 6.4% to 10.0%) (Song et al., 2018). Gout is triggered by urate crystals accumulating in joints, causing pain and inflammation (Riches et al., 2009), and prolonged exposure to high UA levels can result in UA stones and a higher likelihood of kidney disease (Fan et al., 2019). HUA directly or indirectly increases the risk of other chronic diseases (Yadav et al., 2013); several studies have indicated a significant association between HUA and chronic diseases such as obesity, metabolic syndrome, and diabetes (Li et al., 2013; King et al., 2018; Feng et al., 2022; Lee et al., 2022).

Disruptions in purine metabolism and uric acid (UA) excretion contribute to HUA (Qin et al., 2022). Approximately two-thirds of UA is eliminated through the kidneys and the remainder is decomposed by gut microbiota (Chu et al., 2021). When uric acid is secreted into the intestinal lumen, it is rapidly metabolised by microbes in the intestine, such as *Escherichia coli*, *Clostridia* and *Pseudomonas* (Crane, 2013). Once the dysbiosis of gut microbiota occurs, the above pathway would be disrupted, resulting in an imbalance between UA production and excretion, thus causing HUA; this emphasises the essential role of the gut microbiota in the HUA (Lv et al., 2020; Wang et al., 2022). Researches have found that dysbiosis of the gut microbiota is associated with an increased risk of infectious diseases in humans (Cai et al., 2022b; Maciel-Fiuza et al., 2023). Additionally, HUA is a risk factor for infection-related deaths in incident dialysis patients (Yoshida et al., 2020), and is related to patients with sepsis (Sreekanth and A. Maldar, 2022). All evidences indicate that HUA patients are possibly susceptible to bacterial infection via their gut microbiota dysbiosis.

Antimicrobial resistance (AMR) poses an urgent global threat to human health. AMR in the gut is considered an atypical chronic disease. This concern is supported by four main pieces of evidence: (1) the gut microbiome serves as a reservoir for AMR in humans (Anthony et al., 2021); (2) the AMR may persist in the human gut for extended periods and their elimination may prove challenging (Lee et al., 2023); (3) the antimicrobial resistance genes (ARGs) could be acquired by opportunistic bacteria and/or pathogenic bacteria, complicating the treatment of infectious diseases and increasing the risk of transmission and infection (Zhang B. et al., 2021); (4) the severe infections could be caused by resistant bacteria derived from gut microbiota (Libertucci and Young, 2019). Therefore, it is necessary to investigate the landscape of AMR in HUA patients gut, and to assess the risk caused by AMR when administering antibiotic treatment to patients with HUA, it may be necessary to consider the presence of AMR. However, studies thus far have largely focused on alterations in the gut microbiome in the HUA population and associations between microbiome diversity and HUA (de Oliveira and Burini, 2012; Klein et al., 2018; Liang et al., 2022; Wei et al., 2022); the association between the AMR and HUA remains largely unknown.

**Figure 1:**
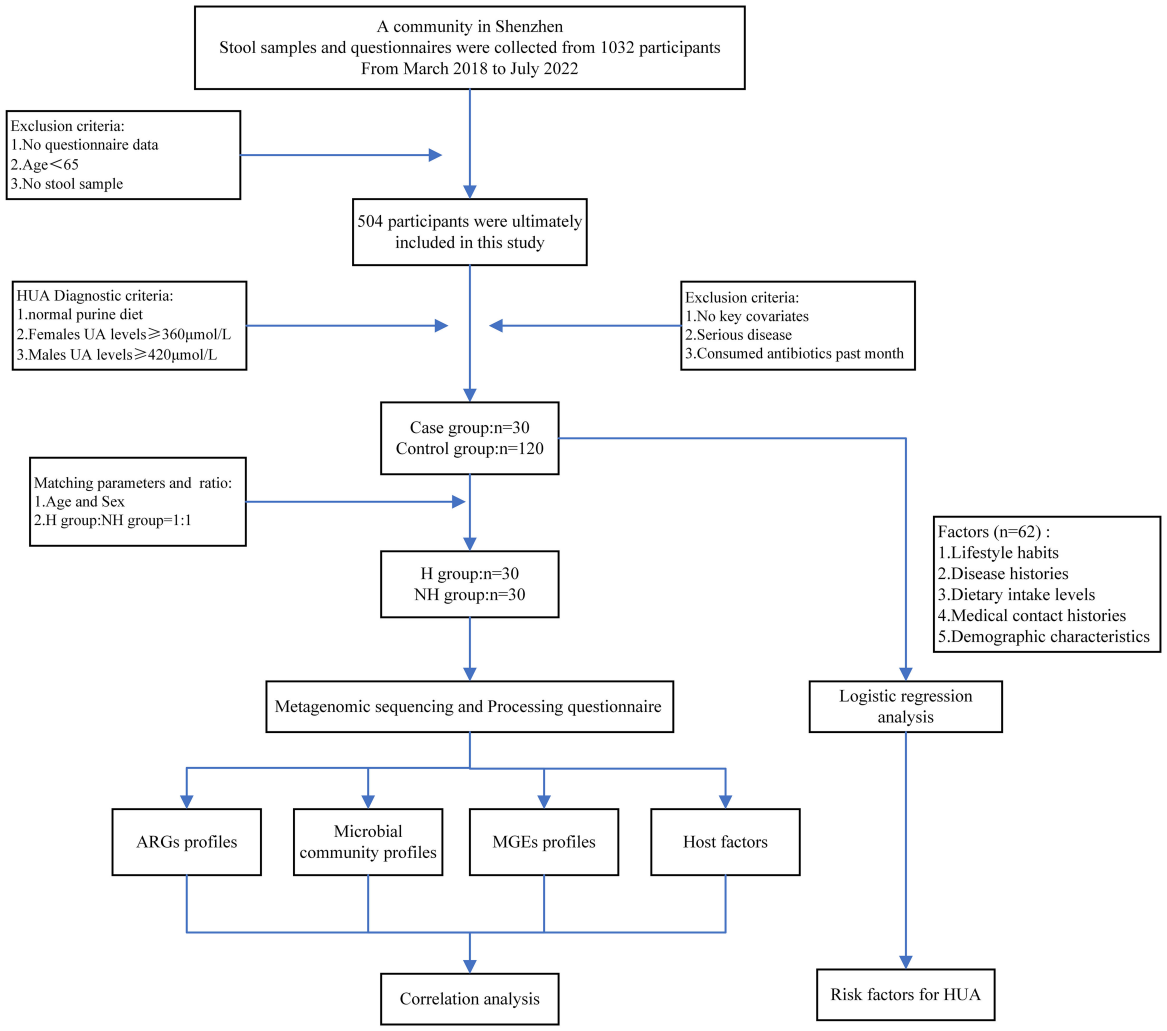
Flowchart of study design.

Hence, this study aimed to depict the landscape of the AMR of 60 elderly people with HUA and non-HUA (30:30) in a large community in Shenzhen, China, and explore its associations with demographics, gut microbiota, resistome (entire ARGs) and mobile genetic elements (MGEs) (**Figure 1**). This study introduces a novel perspective into the existing research on the gut microbiome’s association with HUA.

## Results

### Dietary habits associated with incidence of HUA

**Figure 2:**
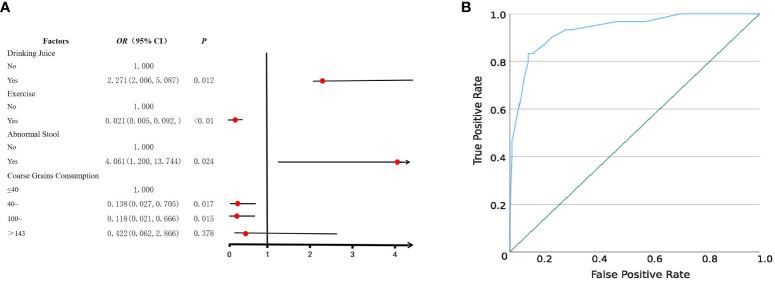
Logistic regression analysis of risk factors for HUA. **(A)** Forest plot reveals the results of multivariate logistic regression analysis. Red dots represent odds ratios (OR). The error bars represent 95% confidence intervals. After screening through univariate analysis (*P* < 0.05) and collinearity-test (VIF < 3), drinking juice, keeping pets, exercise, abnormal stool, coarse grain, and animal offal were included in the univariate analysis. **(B)** The receiver operator characteristic (ROC) curve.

Following a rigorous diagnostic and exclusion process (**Figure 1A**), individuals with HUA and non-HUA individuals were divided into a case group (n=30) and a control group (n=120), respectively. A total of 62 factors in 5 main categories were used for univariate analysis (**Supplementary Table S1**). Significant differences (*P* < 0.05) were observed between the two groups in terms of drinking juice, keeping pets, exercise, abnormal stool, coarse grain and animal offal. Subsequently, 6 factors were used for multivariant logistic analysis via collinearity-test method for excluding confounders. Finally, drinking juice (OR = 2.271, 95%CI: 2.006~5.087, *P* = 0.012) and abnormal stool (OR = 4.061, 95%CI: 1.200~13.744, *P* = 0.024) were risk factors associated with HUA (**Figure 2A**). The adjusted model showed good fit through the Hosmer-Lemeshow test (*χ²* = 2.301, *P* = 0.593), and the receiver operator characteristic (ROC) curve was 0.935 (**Figure 2B**).

### More complex taxonomic diversity in non-hyperuricaemic individuals

**Figure 3:**
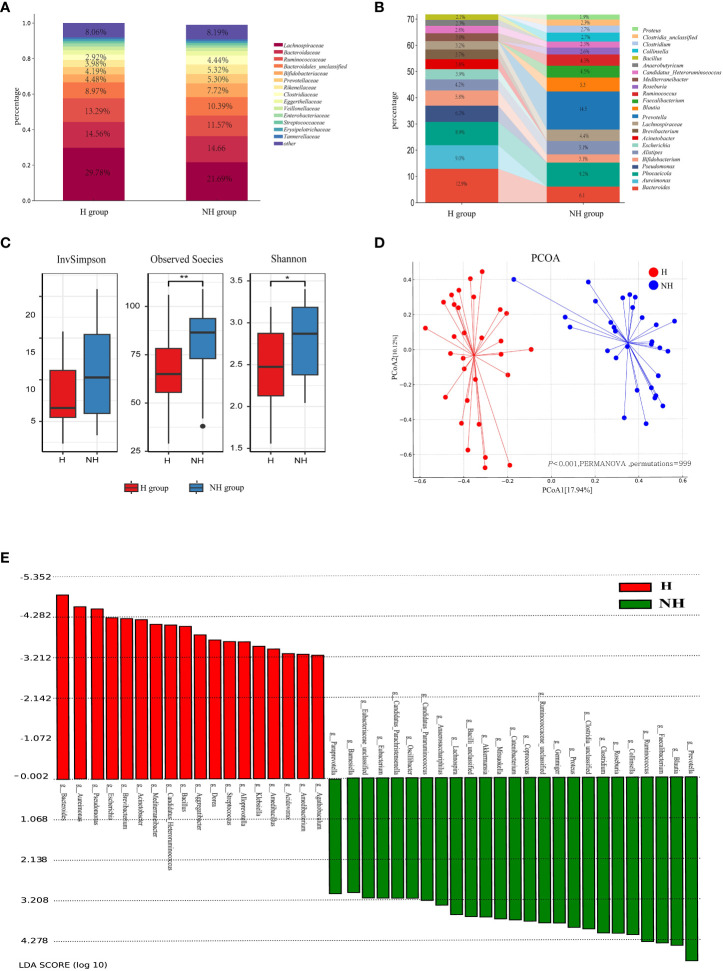
Microbial community profiles between the H group and NH group. **(A)** Distribution of bacteria between the H group and NH group under family level. The proportion of bacteria less than 2% was categorized into ‘other’. **(B)** Distribution of the top 15 bacteria between the H group and NH group under genus level. **(C)** α-diversity. Boxplots show the distribution of data around the median and IQR. *P* values lower than the threshold for significance (* < 0.05, ** < 0.01) were shown. **(D)** β-diversity. Analysis was using Bray Curtis distance and test by PERMANOVA (Bray-Curtis, *P* < 0.05, permutations = 999). **(E)** Linear discriminant analysis (LDA) effect size (LEfSe) analysis indicating the most differential bacteria between the H group and NH group. (LDA SCORE ≥ 103).

*Lachnospiraceae*, *Bacteroidaceae*, and *Ruminococcaceae* were the predominant bacterial families in both groups (**Figure 3A**). The relative abundance of *Bifidobacteriaceae* was higher in the NH group (7.72%) than in the H group (4.48%) (*P* < 0.001, Z-test). *Enterobacteriaceae* accounted for 5.04% in the NH group, while it only accounted for 1.83% in the H group (*P <* 0.001, Z-test). At the genus level, *Prevotella* constituted 14.50% in the NH group but was not listed among the top 15 genera in the H group. Conversely, *Pseudomonas* was ranked fourth in the H group but not in the NH group (**Figure 3B**). These data indicate that the bacterial composition at the genus level in the two groups was distinct.

Subsequently, the diversity and similarity of microbial communities at the genus level in the H group and NH group were analysed using α-diversity indices (Shannon, InvSimpson, Observed species) and β-diversity analyses (principal coordinate analysis [PCoA]). The α-diversity in the H group (Shannon: median 2.47, interquartile range [IQR]: 2.08–2.86; Observed species: median 65.00, IQR: 54–77.5) was significantly lower than that in the NH group (Shannon: median 2.87, IQR: 2.34~3.18, *P =* 0.020, U-test; Observed species: median 86.50, IQR: 69–93.5, *P =* 0.004, t-test, **Figure 3C**). Moreover, the microbial community composition between the H and NH groups was significantly different (*P <* 0.001, permutational multivariate analysis of variance (PERMANOVA), Bray-Curtis; **Figure 3D**).

To better explore the potential biomarkers for HUA, we employed Linear Discriminant Analysis Effect Size (LEfSe) to identify specific genera between the two groups. The results indicated 20 significantly different bacterial genera between the two groups (**Figure 3E**). *Bacillus*, *Bacteroides*, *Escherichia*, *Klebsiella*, *Pseudomonas*, and *Streptococcus* were enriched in the H group, whereas *Catenibacterium*, *Clostridium*, *Collinsella*, *Coprococcus*, *Faecalibacterium*, *Gemmiger*, *Lactobacillus*, *Mitsuokella*, *Prevotella*, *Roseburia*, *Ruminococcus*, *Blautia*, *Lachnospira*, and *Proteus* were enriched in the NH group.

### Higher abundance of ARGs in hyperuriacaemic individuals

**Figure 4:**
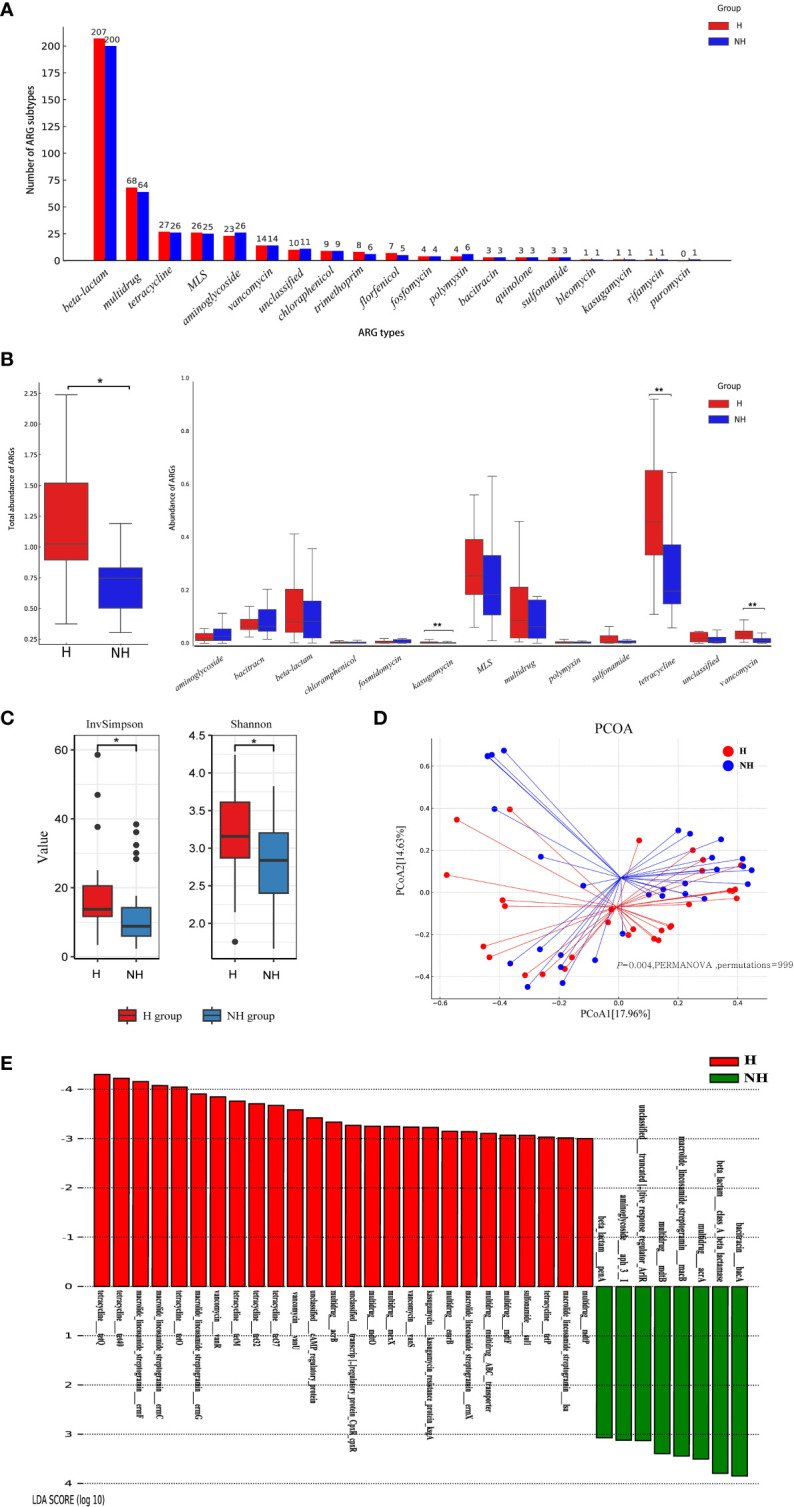
Composition, diversity and specificity of ARGs between the H group and NH group. **(A)** Number of ARG types and subtypes. The horizontal axis represents ARG types, and the vertical axis represents ARG subtypes. **(B)** Abundance of ARG types. Boxplots show the distribution of data around the median and IQR. *P* values lower than the threshold for significance (* < 0.05, ** < 0.01) were shown. **(C)** α-diversity. **(D)** β-diversity. Analysis was using Bray Curtis distance and test by PERMANOVA (Bray Curtis, *P* = 0.004, permutations = 999). **(E)** LEfSe analysis indicating the most differential ARGs between the H group and NH group (LDA SCORE ≥ 10^3^). MLS, Macrolide-Lincosamide-Streptogramin.

The entire ARGs in gut was called resistome. A total of 18 ARG types (417 subtypes) and 19 ARG types (406 subtypes) were identified in the H and NH groups, respectively (**Figure 4A**). In general, 102 ARG subtypes were unique to the H group and 91 to the NH group, and 315 subtypes were shared between the two groups (**Supplementary Figure S1**). Although there were certain commonalities, the distribution patterns of resistome across samples were not consistent (**Supplementary Figure S2**). Total abundance of resistome in the H group (median: 1.10, IQR: 0.93–1.76) was significantly higher than that in the NH group (median: 0.76, IQR: 0.52–1.41, *P* = 0.039, U-test, **Figure 4B**). Specifically, the abundance of tetracycline resistance genes in the H group (median: 0.46, IQR: 0.33–0.65) was significantly higher than in the NH group (median: 0.20, IQR: 0.15–0.37, *P* < 0.001, U-test). Similarly, vancomycin resistance genes in the H group (median: 0.03, IQR: 0.02–0.05) was significantly higher than in the NH group (median: 0.01, IQR: 0.004–0.02, *P* < 0.001, U-test, **Figure 4B**). Furthermore, tetracycline resistance genes were the predominant ARGs, comprising 31.66% and 18.18% in the H and NH groups, respectively (**Supplementary Figure S3**).

We further analysed diversity of resistome between two groups using α-diversity and β-diversity. The α-diversity was significantly greater in the H group (Shannon, median: 3.13, IQR: 2.83–3.62; InvSimpson, median: 13.47, IQR: 11.47–20.67) than that in NH group (Shannon, median: 2.80, IQR: 2.37–3.20, *P* = 0.030, t-test; InvSimpson, median: 8.37, IQR: 6.51–22.98, *P* = 0.040, U-test, **Figure 4C**). Furthermore, different compositions of resistome were observed between the two groups (*P* = 0.004, PERMANOVA, permutations = 999, **Figure 4D**).

LEfSe was used to explore the specific differences in ARG subtypes between the two groups. The results showed that 25 and 8 ARG subtypes were significantly enriched in the H and NH groups, respectively (**Figure 4E**). Specifically, tetracycline resistance genes (*tetQ*, *tet40*, *tetO*, *tetM*, *tet32*, *tet37*, and *tetP*), macrolide-lincosamide-streptogramin (MLS) resistance genes (*ermF*, *ermC*, *ermG*, *ermX*, and *lsa*), vancomycin resistance genes (*vanR*, *vanU* and *vanS*), and several multidrug resistance genes were significantly present in the H group (**Figure 4E**).

More ARGs associated with potential pathogens in hyperuriacaemic individuals.

**Figure 5:**
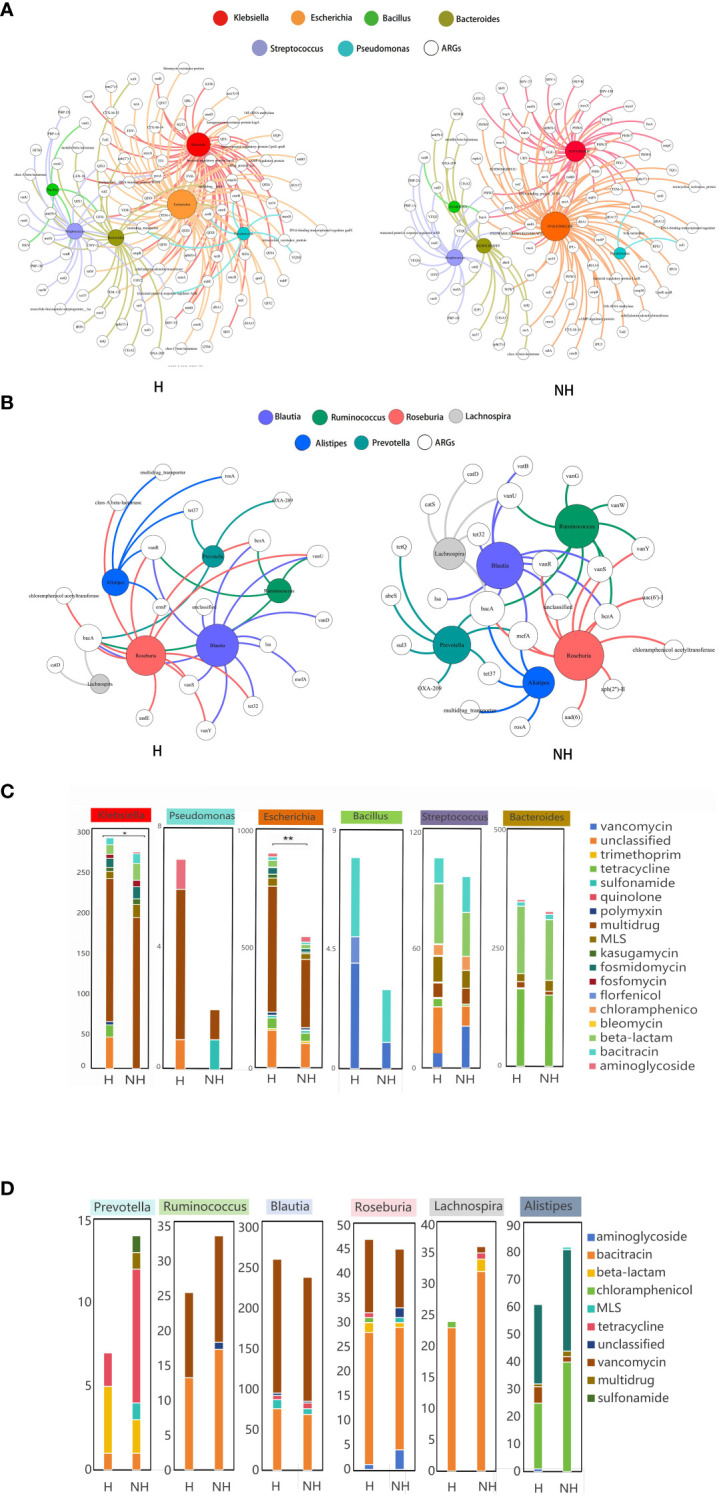
Host distribution of ARGs based on assembly contigs. **(A)** Network analysis between ARGs and potential pathogens based on assembly contigs. **(B)** Network analysis between ARGs and non-pathogens based on assembly contigs. **(C)** The distribution of the number of ARG subtypes in potential pathogens. **(D)** The distribution of the number of ARG subtypes in non-pathogens. *P* values lower than the threshold for significance (* < 0.05, ** < 0.01) were shown.

A total of 9,265 assembled contigs carrying ARGs was obtained from all metagenomics data, in which the 3,957 and 3,835 ARG subtypes were identified in the H and NH group, respectively, after excluding 1,474 contigs without exactly genus information (**Supplementary Table S2**). We further determined the ARGs subtypes in selected six potential pathogens and six non-pathogens that associated with HUA from LEfSe analysis. Total of non-duplicated ARG subtypes in potential pathogens in genus level including *Klebsiella*, *Escherichia*, *Bacteroides*, *Streptococcus*, *Pseudomonas*, and *Bacillus* possessed higher number in H group than those in NH group (175 vs. 147) (**Figure 5A**), and similar and opposite results were found in non-assembly data (128 in H group vs. 47 in NH group) (**Supplementary Figure S4**) and non-pathogens including *Prevotella*, *Ruminococcus*, *Blautia*, *Roseburia*, *Lachnospira*,and *Alistipe* (35 in H group vs. 47 in NH group) (**Figure 5B**).

Furthermore, we also determined the number of ARG subtypes in pathogens and non-pathogens in H and NH group. We found that all pathogens in H group harbour significant higher number of ARG subtypes than those in NH group, especially in *Escherichia* genus (899 Vs. 550, *P <* 0.01, Z-test) (**Figure 5C**). However, the number of ARG subtypes in non-pathogens in H group was similar or lower than those in NH group (**Figure 5D**). In addition, multi-drug resistance genes were likely appeared in *Escherichia*, and the florfenicol resistance genes were found only in *Bacillus* in the H group. Above data indicates that potential pathogens harbour a greater diversity and number of ARGs in HUA individuals.

### Correlations between ARGs and UA

**Figure 6:**
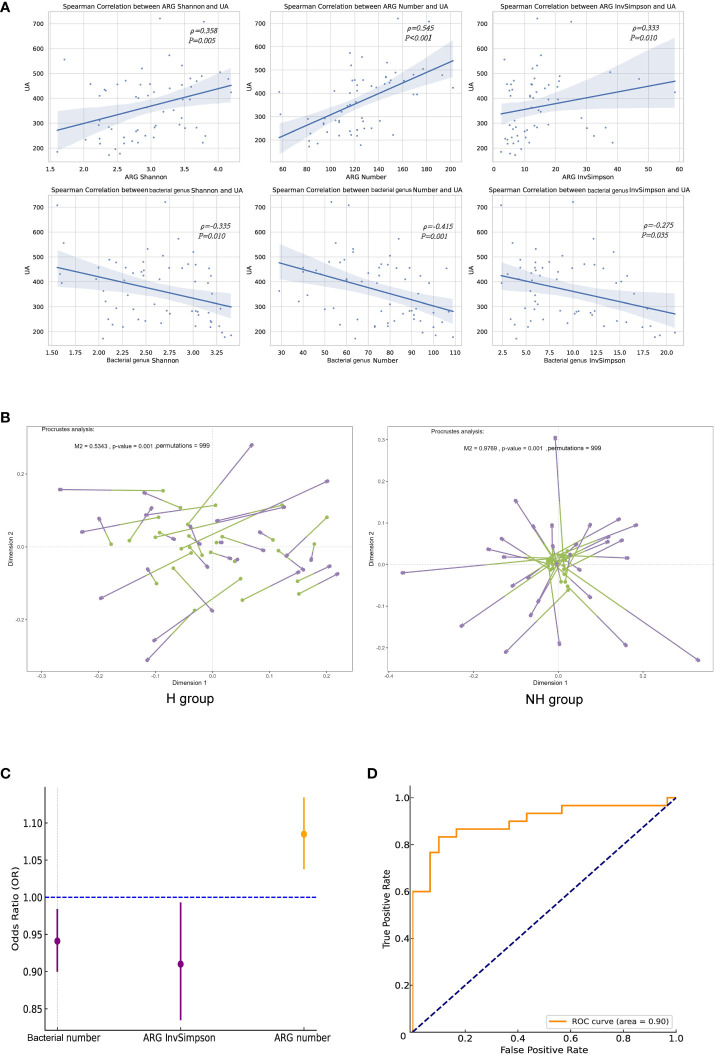
Correlation analyses between ARGs and UA. **(A)** Spearman correlation analysis. **(B)** Procrustes analysis. Longer lines between two dots indicate greater discordance between microbials and ARGs. Significant correlations (999 permutations) were detected in all comparisons. **(C)** Forest plot. The error bars represent 95% confidence intervals. **(D)** The ROC curve.

We further revealed that the ARG number (*ρ* = 0.358, *P* = 0.005), Shannon index (*ρ* = 0.24, *P* < 0.001) and InvSimpson index (*ρ* = 0.333, *P* = 0.010) were significantly correlated with the UA levels; however, they were negatively correlated with gut microbiota (**Figure 6A**). Procrustes analysis demonstrated a strong consistency of ARGs and gut microbiota in the H group (M^2^ = 0.534, *P* = 0.001, permutations = 999), but not in the NH group (M^2^ = 0.977, *P* = 0.001, permutations = 999, **Figure 6B**).

Logistic regression analysis was performed to investigate the association between the ARGs and HUA. After excluding collinearity factors, age, sex, BMI, education, household income, exercise, basal metabolic rate (BMR), gut bacterial indicators (Shannon, number, and InvSimpson), and ARG indicators (Shannon, number, and InvSimpson) were included in our model using a forward selection method. We found that ARG number (OR = 1.090, 95%CI: 1.04–1.13, *P* < 0.001) and ARG InvSimpson (OR = 0.910, 95%CI: 0.836–0.992, *P* = 0.031)/gut bacterial number (OR = 0.941, 95%CI: 0.901–0.983, *P* = 0.006) were positively and negatively associated with HUA, respectively (iterated three times, **Figure 6C**). The goodness and reliability of this model were confirmed using the Hosmer–Lemeshow test (*χ²* = 8.586, *P* = 0.378 > 0.05) and diagnostic receiver operating characteristic (ROC) curve area (0.900) to be satisfactory after adjustment, as evidenced by and the combined with 0.900 value (**Figure 6D**).

### Susceptibility of HUA individuals to ARGs

**Figure 7:**
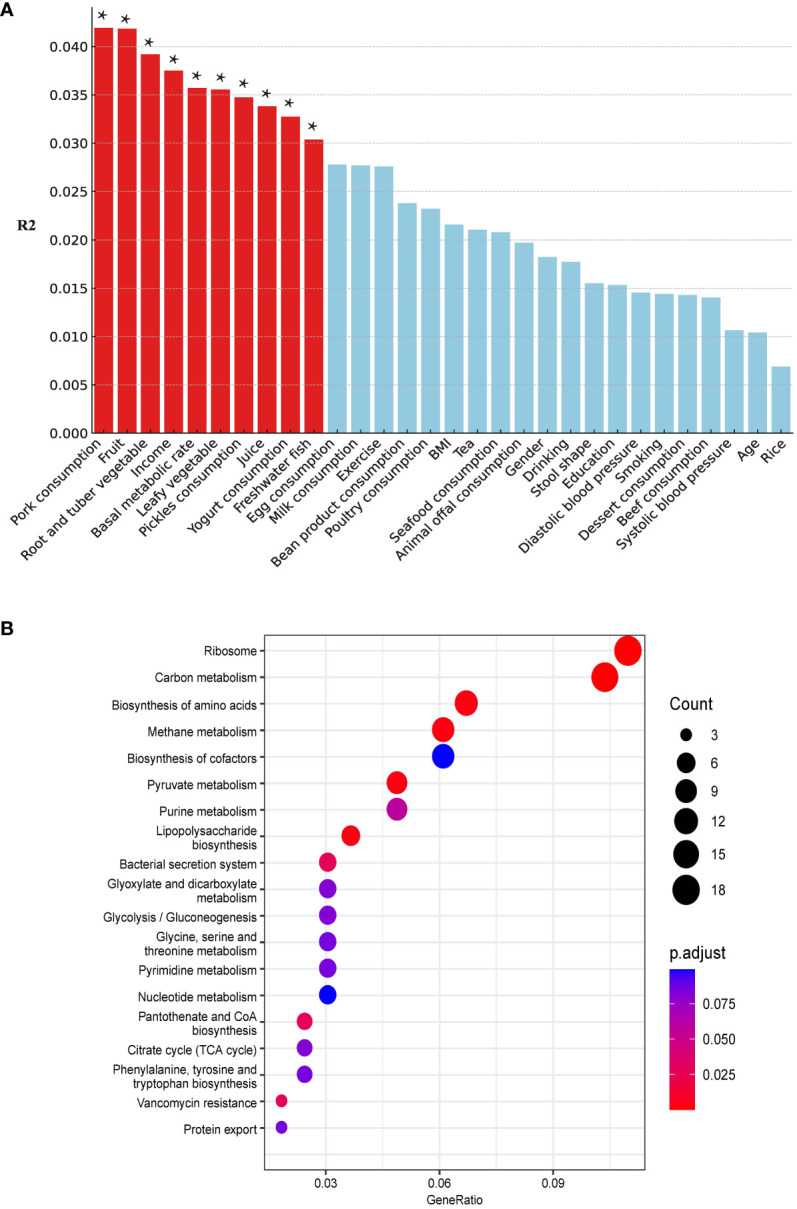
Host associated factors on gut resistome. **(A)** Effect size of host factors on gut resistome were calculated by PERMANOVA (permutations = 999). *P* values lower than the threshold for significance (* < 0.05) were represented as red bars. **(B)** Differential pathways between the H and NH groups. The horizontal axis ‘GeneRatio’ represents the proportion of different genes within a given pathway relative to the total number of different genes. The p.adjust values are Bonferroni-corrected p-values, indicating the statistical significance of the enrichment. The size of the circles correlates with the count of different genes associated with corresponding pathway.

We included 5 demographic factors, 5 physiological factors, and 20 dietary intake levels to investigate the potential factors that may affect the differences in ARGs between the two groups. In total, 10 factors (8 of the 10 factors were associated with dietary consumption) were found to be significantly associated with the ARGs (**Figure 7A**). Pork consumption had the highest explanatory power (*R²* = 0.04, PERMANOVA, *P* = 0.014). We conducted a similar separate analysis for the H and NH groups. Our data showed more factors contributing to the effects on the ARGs in the H group (eight in the H group and three in the NH group) (**Supplementary Figure S5**), especially diet-related factors, associated with the ARGs in HUA individuals, indicating a higher complexity.

Kyoto Encyclopaedia of Genes and Genomes (KEGG) pathway analysis revealed differentially enriched genes between the two groups and indicated different microbial compositions and metabolic functions between the two groups (H vs. NH, **Figure 7B**). The differentially genes between the H and NH groups were found to be enriched in pathways related to ARGs, amino acid biosynthesis and metabolism, phenylalanine, tyrosine, tryptophan biosynthesis, and energy metabolism. This emphasizes the differences in microbial composition between the H and NH groups, particularly in functions related to inflammation, immune response, bacterial secretion, and protein export. All these suggest that the microbiota in H group was prone to possess the higher ability of colonisation and pathogenesis in gut.

We further analysed virulence-associated genes in these two groups and identified 2682 and 2705 VGs in the H and NH groups, respectively (**Supplementary Figure S6**). Genes related to adherence (*P* < 0.001, U-test), antimicrobial activity/competitive advantage (*P* < 0.001, U-test), and exoenzymes (*P* < 0.001, U-test) were significantly upregulated in the H group (**Supplementary Figure S7**). This finding further confirmed our observations in KEGG analyses.

## Discussion

The analysis of risk factors for HUA revealed that drinking juice is one of the risk factors, while exercise and moderate coarse grains are protective factors. These findings are consistent with conclusions drawn from existing literature, highlighting the significant impact of dietary structure and exercise on the occurrence of HUA in elderly individuals (Ashida et al., 2015; Danve et al., 2021). Additionally, this study identified for the first time that abnormal stool was the risk factors for HUA. Abnormal stool characteristics partially reflect the intestinal condition of the subjects, which is closely related to the gut microbiota and indicates a possible dysbiosis of microbiota.

Recent studies have highlighted a link between ARGs and various chronic diseases, including diabetes, cirrhosis, and chronic kidney disease (Shamsaddini et al., 2021; Pan et al., 2022; Shuai et al., 2022). A previous study found that gut microbial diversity was negatively correlated with HUA; however, the gut resistome was not investigated (Yang et al., 2021). One study indicated that patients with HUA and with high UA levels have a weakened immune system and are potentially susceptible to pathogenic infections (Yu et al., 2023). Notably, our study found that elderly individuals with HUA had a higher abundance of ARGs in their gut than those without HUA. Furthermore, the abundance of ARGs in individuals with HUA was positively correlated with UA levels and the incidence of HUA. As the resistome in the gut microbiota is considered an atypical chronic disease, a higher abundance of the resistome increases the risk to human health. However, as the number of elderly individuals with HUA increases annually, health risks and socioeconomic burdens become more severe (Maresova et al., 2019). One study discovered that the burden of ARGs increases with human ageing (Tavella et al., 2021). Hence, more attention should be given to elderly individuals with HUA and higher resistome abundance to find an effective way of reducing resistome abundance and associated pathogens in the human gut.

The study identified 20 different species in the two groups, with *Bacillus*, *Bacteroides*, *Escherichia*, and *Klebsiella* being enriched in group H. Uricase, which is responsible for converting purines into urea and UA, is abundant in *Bacillus* (DeBosch et al., 2014). *Escherichia* secretes the key enzyme, xanthine dehydrogenase (XDH), which is crucial for purine oxidative metabolism (Wang et al., 2017). Our study corroborates the findings of a hospital population study, which revealed a significant reduction in gut microbial diversity in patients with HUA, accompanied by a notable increase in *Bacteroides* and a decrease in *Ruminococcaceae* (Liang et al., 2022). In the NH group, bacteria such as *Lactobacillus* and *Clostridium* were enriched. *Lactobacillus* is known to absorb and utilise purines, thereby reducing the intestinal absorption of dietary purines and lowering serum UA levels (Yamada et al., 2016). Additionally, *Clostridium* has been associated with UA decomposition (Yu et al., 2018). The core microbiome of HUA may be related to the gut microbiota, which is enriched in purine metabolism-related proteins, and *Bacteroides* is an important component (Liu et al., 2023). A close relationship between *Klebsiella* and elevated UA levels has been revealed (Yang et al., 2021). Our data on the composition of the gut microbiome in individuals were consistent with the findings of previous studies. Emphasis should be placed on the fact that *Aureimonas*, a common human opportunistic pathogen in the environment (Becker et al., 2022), was enriched in HUA individuals in this study; however, the relationship between *Aureimonas* and HUA remains unclear. A recent study found that hippuric acid derived from *Alistipes indistinctus* promotes intestinal UA excretion to alleviate HUA (Xu et al., 2024). In our study, we also observed that the total relative abundance of *Alistipes* in the H group (126.94) was lower than that in the NH group (150.96). Our results indicated a potential increase in the diversity, number and abundance of ARGs in the gut microbiota of patients with HUA. In addition, bacteria associated with HUA may have elevated levels of these ARGs. Furthermore, Procrustes analysis and logistic regression revealed a synergy between the diversity of ARGs and bacterial populations, which might collectively be associated with UA levels and the occurrence of HUA.

Pork consumption was the predominant factor associated with the gut resistome in individuals with HUA. This may be attributed to Guangdong being one of the largest pork-consuming regions in China. One study revealed high concentrations of tetracycline resistance genes in water samples from the coastal areas of Guangdong (Xu et al., 2019), whereas another revealed high concentrations of multidrug resistance genes in chicken and pork from Guangdong retail markets (Zhang et al., 2018). In the present study, tetracycline and multidrug ARGs were the most abundant subtypes. The above evidence suggests that water, meat, and human guts in Guangdong Province are contaminated with higher ARGs. Separate analyses of the H and NH groups indicated that the factors influencing gut ARGs composition differed under varying HUA conditions. In individuals with HUA, the influencing factors were primarily sex, dietary habits (including juice, rice, and vegetables), and education level. In contrast, in individuals without HUA, the influencing factors were limited to dietary habits, specifically fat consumption and diversity. These results indicate that lifestyle habits and personal characteristics of the subjects could be external factors affecting the variation in their gut ARG composition.

Pathway analysis revealed that in individuals with HUA, genes of the gut microbiota were enriched in inflammation, immunity, and bacterial survival, including vancomycin resistance, lipopolysaccharide biosynthesis, bacterial secretion system, and amino acid metabolism. This aligns with the higher abundance of genes related to adherence, antimicrobial activity, and exoenzymes in individuals with HUA. Additionally, the more complex relationship between MGEs and ARGs/microbes in individuals with HUA indicated a higher frequency of ARGs transmission in these individuals. These data suggest that the microbiota of individuals is associated with higher antimicrobial resistance and pathogenicity, which may increase the risk to human health. Combined with our findings, dietary habits may vary the composition of the gut microbiota and reconstruct the gut resistome, resulting in an imbalance of microbiota with the excretion of UA and potentially raising UA levels, which may initiate or exacerbate HUA.

Our study has some limitations. First, we focused exclusively on the elderly population, which may not be a representative of the entire population. Second, as an observational study, our findings establish a correlation rather than causality; further studies are required to elucidate the causal relationships.

## Conclusion

Our study offers a comprehensive overview of the gut AMR in the elderly population and presents novel insights into the relationship between AMR and HUA. It also suggests a positive association between the gut AMR and host-associated factors. These findings highlight that individuals with HUA possess a higher risk of AMR, and potential intervention approaches are needed to be applied to reduce AMR in HUA individuals gut based on significant host-associated factors.

## Methods

### Study design and participants

A flowchart of the study design is shown in **Figure 1**. A total of 1,032 volunteers were recruited through advertising posters and face-to-face introductions in a large community in Shenzhen City from March 1^st^ 2018 to July 31^st^ 2022. Each volunteer was assigned a unique number to avoid duplicate enrolment. Subsequently, participants provided stool samples and completed a questionnaire. Individual consent forms were translated into Mandarin, and consent was obtained from all volunteers. We did not recruit volunteers for whom consent could not be obtained. All participants retained the right to withdraw from the study at any stage.

Given the study’s focus on elderly people; we applied the following exclusion criteria: age less than 65 years, absence of stool samples, and lack of questionnaire data. After exclusion, 504 participants were included in this study. Subsequently, subjects were included based on HUA diagnostic criteria: 1) a normal purine diet, 2) blood uric acid (UA) levels exceeding 360 μmol/L for females and 420 μmol/L for males, respectively (Luo et al., 2022). We also excluded individuals who were missing key covariate data such as age, gender, BMI, smoking status, alcohol consumption, education level, and income level, and also who had serious illnesses like other gastrointestinal disorders, HIV, or cancer and consumed antibiotics within the past month. Finally, 30 participants with HUA were included in the case group. A matching ratio of 1:4 was used to randomly select participants (n = 120, 1:4) without HUA as the control group. After 1:1 matching by age and sex, the 30 participants in the case group and the 120 participants in the control group were respectively divided into the H group and the NH group for subsequent analysis.

### Metadata collection and process

Demographics, lifestyle, medical history, and physical activity data were collected using a questionnaire. Participants’ habitual dietary intake was assessed using a food frequency questionnaire that recorded their food consumption over a week. Trained nurses from the community health centre measured the weight, height, waist circumference, hip circumference, and blood pressure. All data were entered using EpiData Version 3.1 with a double-entry method.

### DNA extraction and metagenomic sequencing

All stool samples were placed in a low-temperature transportation box and promptly sent to the laboratory (within 8 h) and were stored at -80°C until they were ready for genomic DNA extraction. The genomic DNA was extracted using Tiangen Magnetic Beads (Beijing, China). The purity and integrity of the extracted DNA were evaluated by 1% agarose gel electrophoresis. The DNA was quantified using the Qubit^®^ dsDNA Assay Kit by Qubit^®^ 2.0 Fluorometer (Thermo Fisher Technologies, CA, USA). Approximate 1 μg of DNA was used for library construction using the NEBNext^®^ Ultra DNA Library Prep Kit for Illumina (NEB, CA, USA). Preliminary quantification was performed using Qubit 2.0 after library construction, and subsequently, the insert size of the DNA library was checked using Agilent 2100 (Agilent, CA, USA). The effective concentration of the library was accurately quantified using the Q-PCR method (the effective concentration of the library > 3 nM). Paired-end sequencing reads were generated using an Illumina NovaSeq platform (Illumina, CA, USA) by Novogene Co., Ltd. All metagenomic sequence data had been uploaded to the public NCBI sequence read archive (SRA) database under the BioProject number PRJNA1059748.

### Metagenomic analysis

Raw reads were quality-filtered using fastp (Chen et al., 2018) to remove sequences with a quality value > 38 or ambiguous nucleotides > 10. In cases where there was potential host contamination, default alignment was performed using Bowtie2 software (version 2.2.4, http://bowtie-bio.sourceforge.net/bowtie2/index.shtml) with the following parameters (Karlsson et al., 2013): –end-to-end, –sensitive, –I 200, –X 400. The reads with a match consistency ≥ 90% were filtered out. After quality filtering, approximately 10 GB of clean data were obtained for each sample. MetaPhlAn version 4.0.4 (17 Jan 2023) was used to analyse the relative abundance data at the family and genus levels (Truong et al., 2015). The abundances of ARGs, virulence genes (VGs), and MGEs were calculated using ARGs-SOP (Version 2.0) with default parameters using the SARG.2.2, VFDB, and Mobile Genetic Element Databases, respectively (Liu et al., 2022). To identify the hosts of antibiotic resistance genes, we used MEGAHIT version1.0.4-beta to assemble the data (Nielsen et al., 2014), and the contigs longer than 500 bp were kept for subsequent analyses. The filtered contigs were annotated for species using Kraken2 and we used DIAMOND to search the protein sequences against the SARG.2.2 database to identify ARGs containing contigs. The relative abundance of the KEGG pathway was determined using HUMANN3 (Franzosa et al., 2018).

### Statistical analysis

For risk factors analyses, univariate analysis was conducted using the chi-square test or Fisher’s exact test (expected frequency < 5, use Fisher’s exact test; expected frequency > 5, use chi-square test). A collinearity-test was performed on the variables to select those with a variance inflation factor (VIF) < 3 for subsequent multivariate logistic regression analysis (VIF < 3 indicates no collinearity). Variables with *P* < 0.05 and VIF < 3 in previous analyses were further included in multivariable logistic regression model (enter, *P* < 0.05; excluded, *P* > 0.1, forward selection). We used the Wilcoxon rank-sum test (Mann–Whitney *U*-test) or t-test (depending on whether the data were normally distributed) to compare the number of observed features (i.e. richness), InvSimpson (i.e. richness), and Shannon index (i.e. diversity) (Anderson, 2001). The Z-test was used to compare the proportions of microbiota. We conducted PCoA (based on Bray–Curtis distances) to explore the similarities and differences in microbiota/ARGs/MGEs between the H and NH groups (Nocelli et al., 1999). LEfSe was used to distinguish differences in microbiota and ARGs between and within the H and NH groups (Segata et al., 2011). We also used Spearman’s correlation analysis to evaluate the linear associations between ARGs richness and diversity, and UA levels. All analyses were performed in R v3.6.1 (R Foundation for Statistical Computing, Vienna, Austria). *P* values < 0.05 were considered statistically significant. Heatmaps were generated using the heatmap package, while most other graphs such as box plot and bar chart were produced using the ggplot2 package (Chen et al., 2020). The α-diversity indices (Shannon, observed species, InvSimpson) of bacterial communities, ARGs, and MGEs for each sample were calculated using vegan package in R (Barberán et al., 2012). The β-diversity analysis was conducted using PCoA based on Bray–Curtis distances via vegan package (Cao et al., 2020), and PERMANOVA was used for statistical analysis (Anderson, 2001). We conducted Procrustes analysis based on Bray–Curtis dissimilarity to investigate the relationship between ARGs and gut microbiota. Network analyses between potential pathogens and ARGs were visualised using Gephi v0.9.2 (Chen et al., 2020).

## Data availability statement

The datasets presented in this study can be found in online repositories. The names of the repository/repositories and accession number(s) can be found here: NCBI SRA, accession PRJNA1059748.

## Ethics statement

The studies involving humans were approved by Ethical approval (R2018021) was granted by the Ethics Committee of the Shenzhen CDC on 19 January 2018. The studies were conducted in accordance with the local legislation and institutional requirements. The participants provided their written informed consent to participate in this study.

## Author contributions

ZhL: Conceptualization, Data curation, Investigation, Methodology, Software, Writing – original draft, Writing – review & editing. YS: Methodology, Supervision, Writing – original draft, Writing – review & editing. YF: Data curation, Methodology, Software, Supervision, Writing – review & editing. DS: Data curation, Software, Writing – review & editing. LL: Supervision, Writing – original draft. ZiL: Conceptualization, Formal analysis, Funding acquisition, Project administration, Resources, Writing – original draft.
