# Supplementary material for: Association of resistome abundance with hyperuricaemia in elderly individuals: a metagenomics study
Source: Front Microbiomes. 2024 Jul 11;3:1384703. doi: 10.3389/frmbi.2024.1384703 (PMC12993550; doi:10.3389/frmbi.2024.1384703)
Supplement: Supplementary file 1 [file DataSheet_1.docx]

**Supplementary**


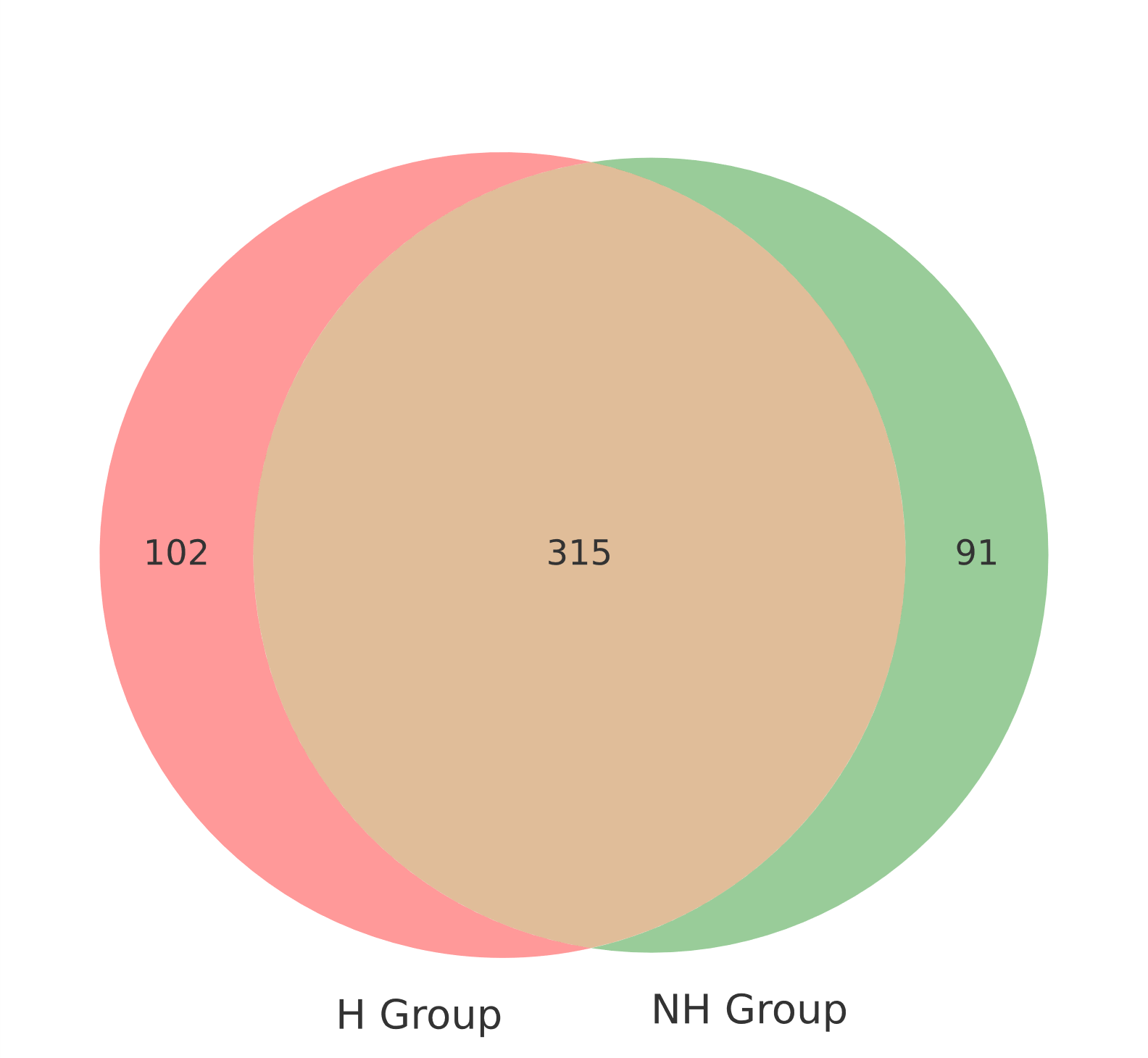


Figure S1. Venn diagram showing shared ARG subtypes among H and NH Groups.


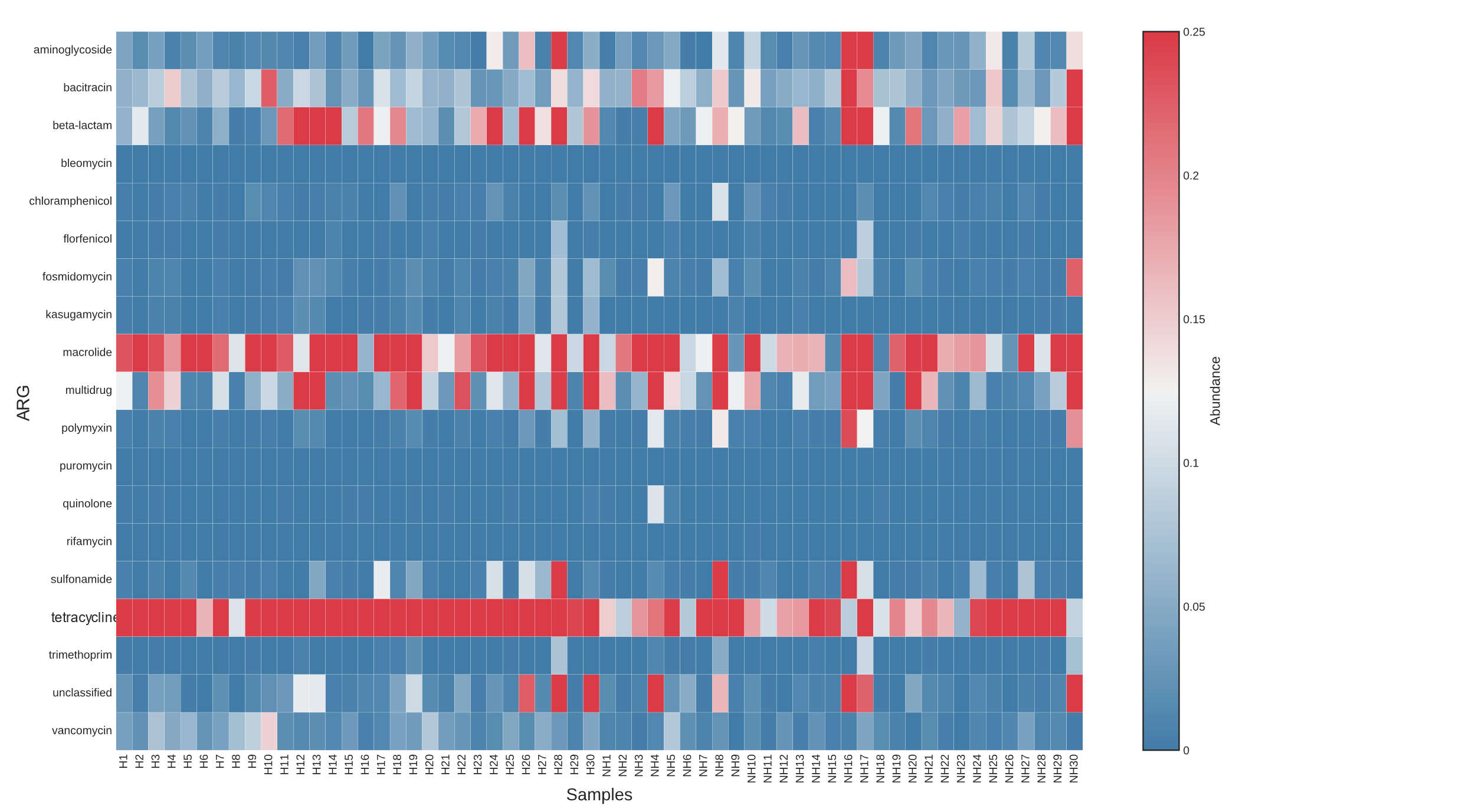


Figure S2. Heatmap of relative abundance of ARG types across 60 samples.


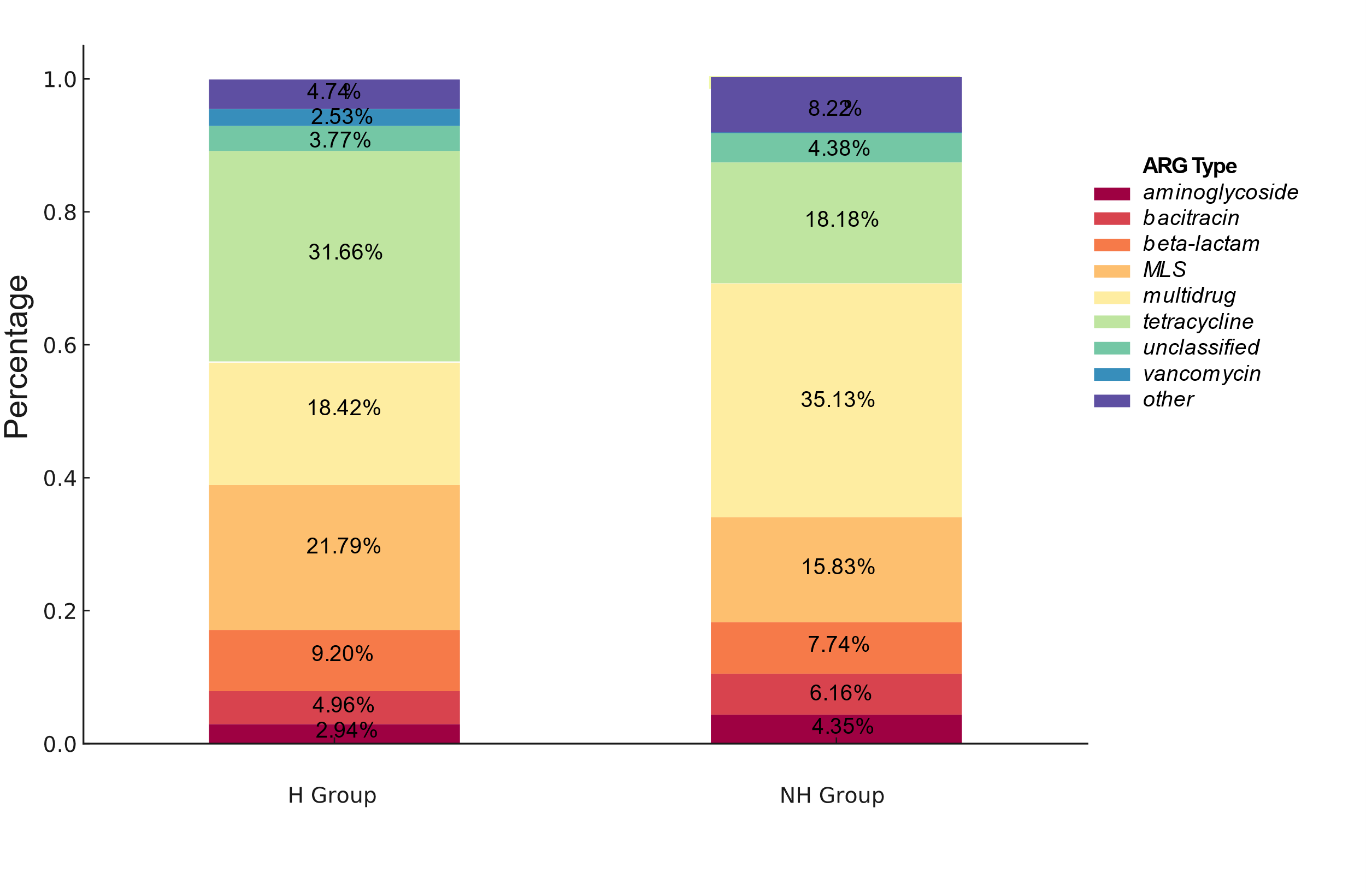


Figure S3. Distribution of ARG type between H and NH groups. ARG type with a proportion less than 2% were categorized as 'other'. MLS, Macrolide-Lincosamide-Streptogramin.


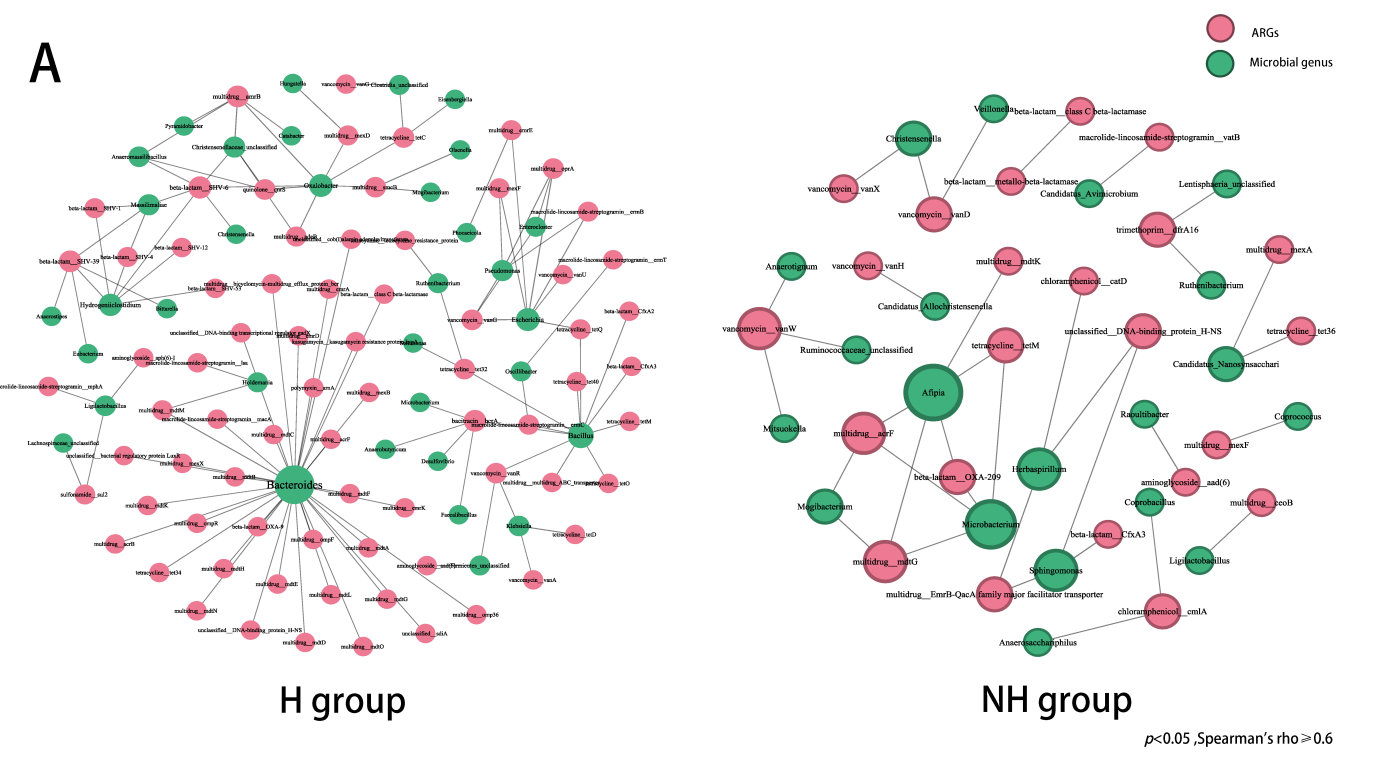


Figure S4. Co-occurrence patterns among microbials and ARGs based on unassembled metagenomic data. Nodes represent corresponding items and lines connecting nodes indicate significant pairwise correlation based on Spearman correlation analysis and FDR adjustment (P < 0.05, 0.05 and 0.01, rho ≥ 0.6, 0.6 and 0.8, respectively).


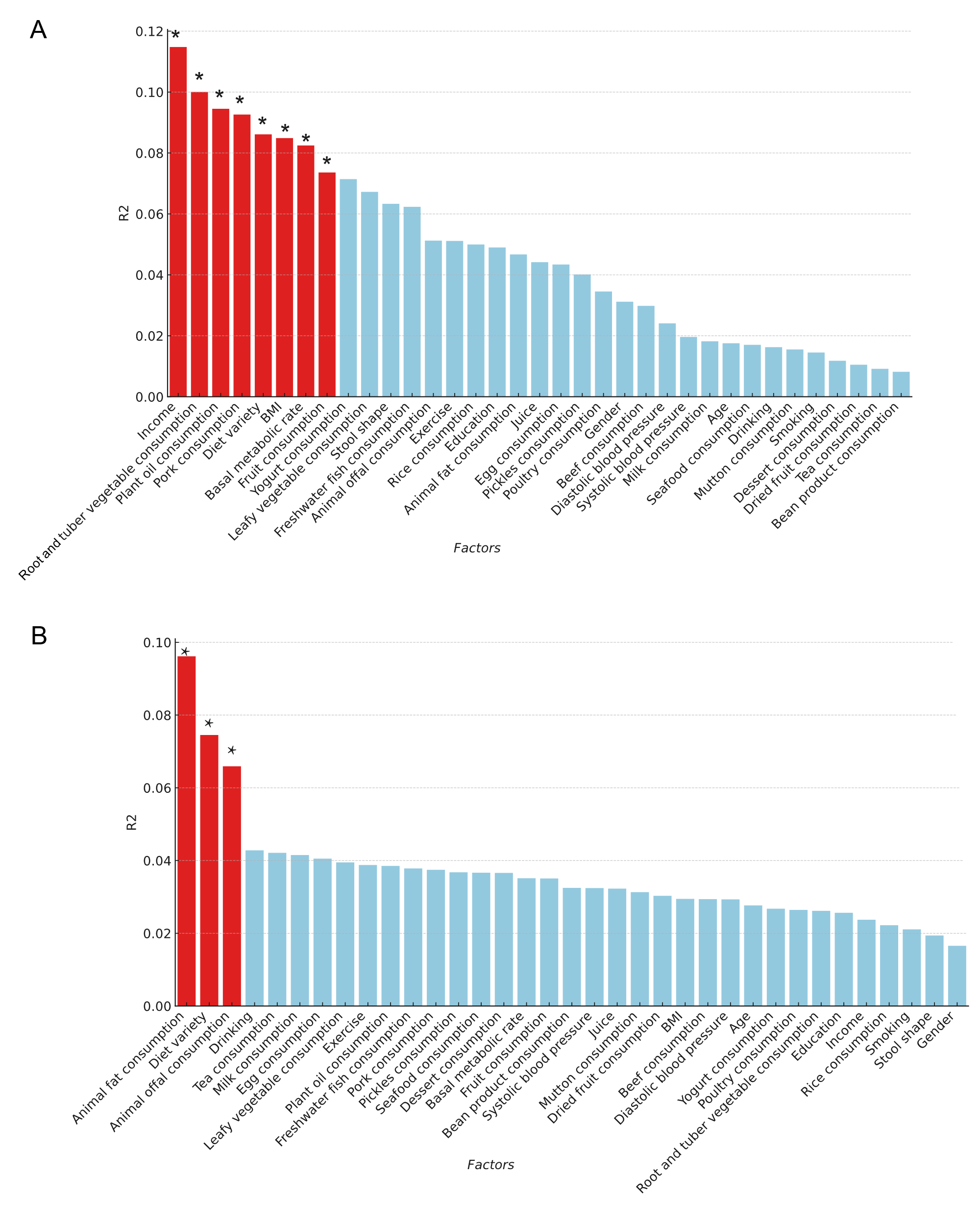


Figure S5. Effect size of host associated factors on gut resistome. (A) HUA. (B) non-HUA. The R2 was calculated by PERMANOVA (permutations = 999). *P* values lower than the threshold for significance (* < 0.05) were represented as red bars.


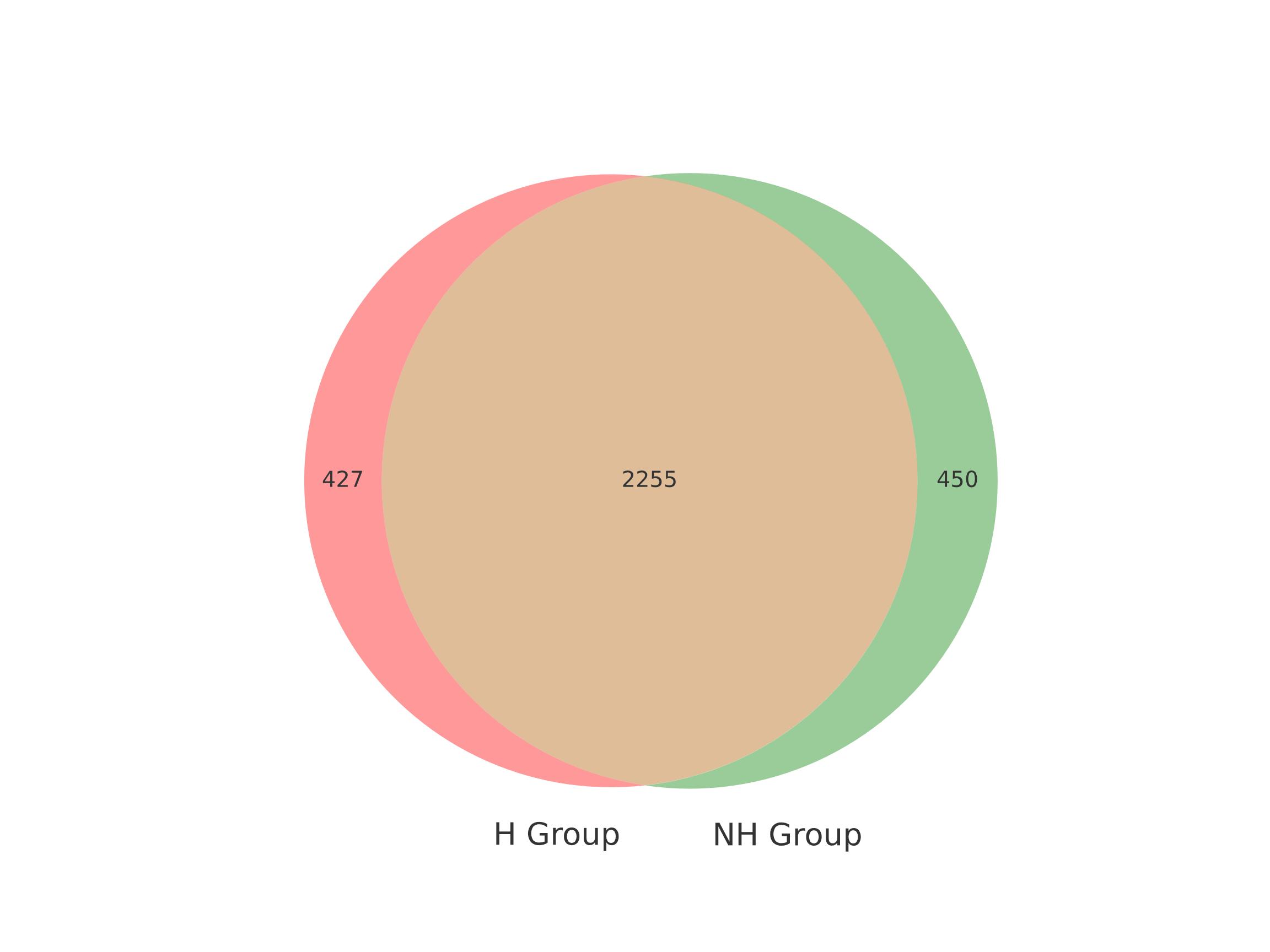


Figure S6. Venn diagram showing shared VGs among H and NH Groups.


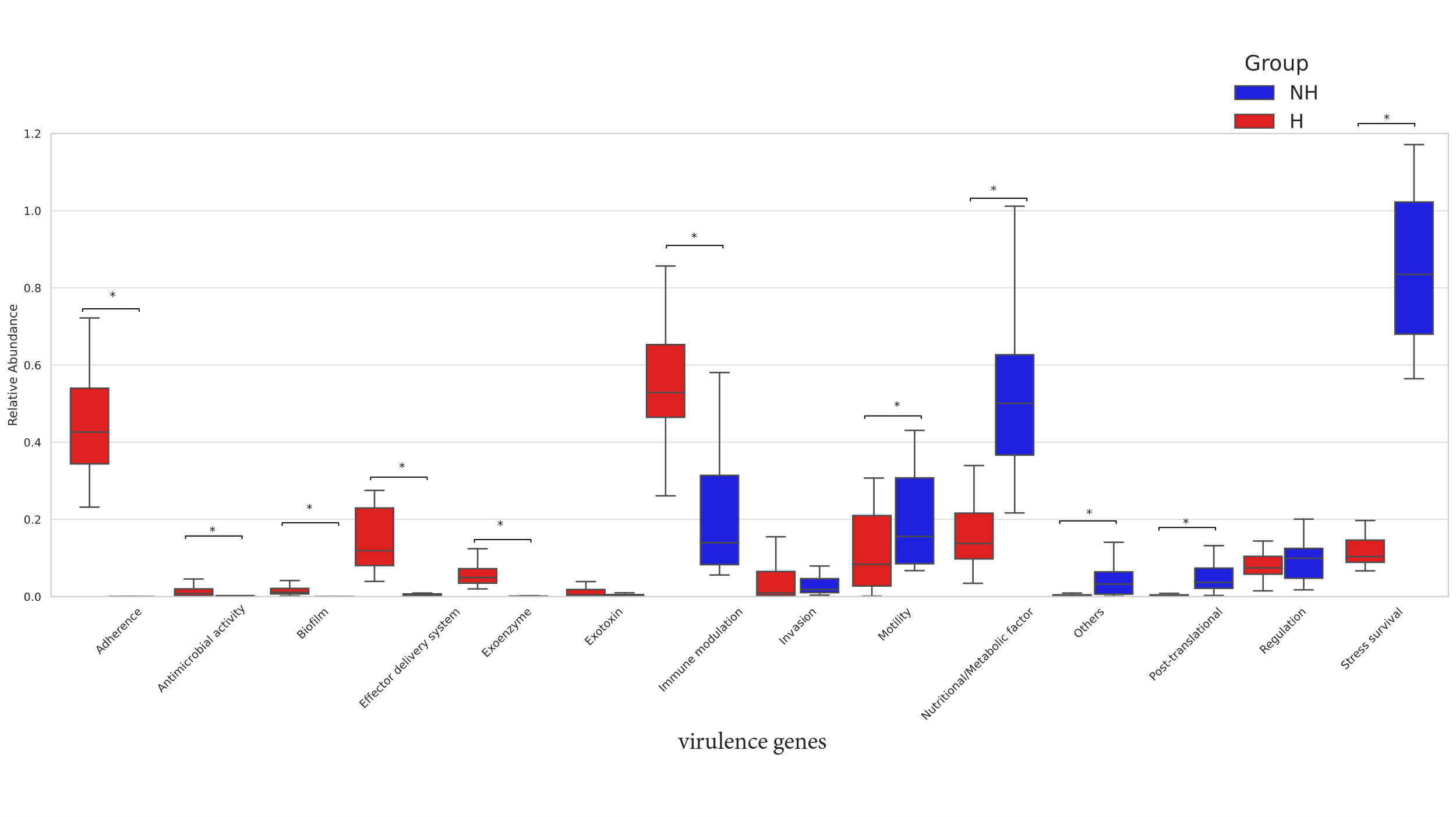


Figure S7. The relative abundance of virulence genes (VGs). Boxplots show the distribution of data around the median and IQR. *P* values lower than the threshold for significance (* < 0.05, ** < 0.01) were shown.
