# Supplementary material for: Association of resistome abundance with hyperuricaemia in elderly individuals: a metagenomics study
Source: Front Microbiomes. 2024 Jul 11;3:1384703. doi: 10.3389/frmbi.2024.1384703 (PMC12993550; doi:10.3389/frmbi.2024.1384703)
Supplement: Supplementary file 2 [file Table_1.docx]

**Table S1. Univariate analysis of the risk factors for HUA [*N* (%)]**

| **Variable** | **Control**  **(n = 120)** | **Case**  **(n = 30)** | **χ2** | | ***P*** |
| --- | --- | --- | --- | --- | --- |
| ***Demographic characteristics*** | | | | | |
| **gender** |  |  | 2.973 | | 0.085 |
| Male | 41 (34.2) | 16 (53.3) |  | |  |
| Female | 79 (65.8) | 14 (46.7) |  | |  |
| **age** |  |  | 2.564 | | 0.109 |
| ≥ 70 | 62 (51.7) | 21 (70.0) |  | |  |
| < 70 | 58 (48.3) | 9 (30.0) |  | |  |
| **education level** |  |  | 0.480 | | 0.787 |
| Primary | 36 (30.0) | 7 (23.3) | |  |  |
| Middle | 55 (45.8) | 15 (50.0) | |  |  |
| College | 29 (24.2) | 8 (26.7) | |  |  |
| **BMI (kg/m2)** |  |  | | ------ | 0.402* |
| 18.5~ | 52 (43.3) | 10 (33.3) | |  |  |
| < 18.5 | 5 (4.2%) | 2 (6.7) | |  |  |
| ≥ 24 | 63 (52.5) | 18 (60.0) | |  |  |
| **ethnicity** |  |  | | ------ | > 0.999* |
| Han Chinese | 119 (99.2) | 28 (93.3) | |  |  |
| Other | 1 (0.8) | 2 (6.7) | |  |  |
| **income (RMB)** |  |  | | 1.439 | 0.487 |
| ≤ 2000 | 41 (34.2) | 7 (23.4) | |  |  |
| 2001~ | 58 (48.3) | 16 (53.3) | |  |  |
| 6001~ | 21 (17.5) | 7 (23.3) | |  |  |
| **sampling time** |  |  | | 0.000 | > 0.999 |
| 2018~2019 | 44 (36.7) | 11 (36.7) | |  |  |
| 2019~2021 | 36 (30.0) | 9 (30.0) | |  |  |
| 2021~2022 | 40 (33.3) | 10 (33.3) | |  |  |
| ***Disease histories*** | | | | | |
| **hypertension** |  |  | | 2.079 | 0.149 |
| No | 72 (60.0) | 13 (43.3) | |  |  |
| Yes | 48 (40.0) | 17 (56.7) | |  |  |
| **hyperlipidemia** |  |  | | 0.024 | 0.877 |
| No | 96 (80.0) | 25 (83.3) | |  |  |
| Yes | 24 (20.0) | 5 (16.7) | |  |  |
| **diabetes** |  |  | | ------ | 0.289* |
| No | 96 (80.0) | 27 (90.0) | |  |  |
| Yes | 24 (20.0) | 3 (10.0) | |  |  |
| **coronary heart disease** |  |  | | ------ | 0.766* |
| No | 103 (85.8) | 27 (90.0) | |  |  |
| Yes | 17 (14.2) | 3 (10.0) | |  |  |
| **myocardial infarction** |  |  | | ------ | > 0.999* |
| No | 118 (98.3) | 30 (76.7) | |  |  |
| Yes | 2 (1.7) | 0 (0.0) | |  |  |
| **stroke** |  |  | | ------ | > 0.999* |
| No | 114 (95.0) | 29 (96.7) | |  |  |
| Yes | 6 (5.0) | 1 (3.3) | |  |  |
| **biliary tract disease** |  |  | | ------ | > 0.999* |
| No | 105 (87.5) | 27 (90.0) | |  |  |
| Yes | 15 (12.5) | 3 (10.0) | |  |  |
| **nephritis** |  |  | | ------ | > 0.999* |
| No | 117 (97.5) | 30 (100.0) | |  |  |
| Yes | 3 (2.5) | 0 (0.0) | |  |  |
| ***Lifestyle habits*** | | | | | |
| **smoking** |  |  | | 0.772 | 0.380 |
| No | 99 (82.5) | 22 (73.3) | |  |  |
| Yes | 21 (17.5) | 8 (26.7) | |  |  |
| **drinking** |  |  | | 2.713 | 0.100 |
| No | 27 (85.0) | 21 (83.3) | |  |  |
| Yes | 3 (15.0) | 9 (16.7) | |  |  |
| **drinking tea** |  |  | | 0.377 | 0.539 |
| No | 62 (51.7) | 18 (60.0) | |  |  |
| Yes | 58 (48.3) | 12 (40.0) | |  |  |
| **drinking juice** |  |  | | ------ | **< 0.001*** |
| No | 118 (98.3) | 24 (80.0) | |  |  |
| Yes | 2 (1.7) | 6 (20.0) | |  |  |
| **keeping pets** |  |  | | ------ | **0.025*** |
| No | 115 (95.8) | 28 (93.3) | |  |  |
| Yes | 5 (4.2) | 2 (6.7) | |  |  |
| **source of drinking water** |  |  | | ------ | > 0.999* |
| Community | 112 (93.3) | 28 (93.3) | |  |  |
| Bottled water | 8 (6.7) | 2 (6.7) | |  |  |
| **using water filter device** |  |  | | 1.354 | 0.245 |
| No | 44 (36.7) | 7 (23.3) | |  |  |
| Yes | 76 (63.3) | 23 (76.7) | |  |  |
| **boiling drinking water** |  |  | | ------ | > 0.999* |
| No | 3 (2.5) | 0 (0.0) | |  |  |
| Yes | 117 (97.5) | 30 (100.0) | |  |  |
| **regular diet** |  |  | | ------ | < 0.687* |
| No | 5 (4.2) | 2 (6.7) | |  |  |
| Basic | 55 (5.8) | 12 (40.0) | |  |  |
| Very | 60 (50.0) | 16 (53.3) | |  |  |
| **eating frequency** |  |  | | ------ | 0.318* |
| 2/day | 8 (6.7) | 1 (3.3) | |  |  |
| 3/day | 103 (85.8) | 29 (96.7) | |  |  |
| ≥4/day | 9 (7.5) | 0 (0.0) | |  |  |
| **variety of food** |  |  | | ------ | 0.729* |
| Meat-based | 69 (57.5) | 19 (63.3) | |  |  |
| Balanced | 35 (29.2) | 9 (30) | |  |  |
| Vegetarian-based | 16 (13.3) | 2 (6.7) | |  |  |
| **exercise** |  |  | | ------ | **< 0.001*** |
| No | 18 (15.0) | 25 (83.3) | |  |  |
| Yes | 102 (85.0) | 5 (16.7) | |  |  |
| **abNormal stool** |  |  | | ------ | **< 0.001*** |
| No | 90 (78.3) | 10 (33.3) | |  |  |
| Yes | 30 (21.7) | 20 (66.7) | |  |  |
| ***Medical contact histories*** | | | | | |
| **surgery** |  |  | | ------ | 0.200***** |
| No | 120 (100.0) | 29 (96.7) | |  |  |
| Yes | 0 (0.0) | 1 (3.3) | |  |  |
| **hospitalization** |  |  | | ------ | 0.583***** |
| No | 115 (95.8) | 30 (100.0) | |  |  |
| Yes | 5 (4.2) | 0 (0.0) | |  |  |
| **history of visiting patients** |  |  | | ------ | > 0.999***** |
| No | 110 (91.7) | 28 (93.3) | |  |  |
| Yes | 10 (8.3) | 2 (6.7) | |  |  |
| **taking dietary supplements** |  |  | | ------ | 0.460***** |
| No | 112 (93.3) | 27 (90.0) | |  |  |
| Yes | 8 (6.7) | 3 (10.0) | |  |  |
| **lipid-lowering medication** |  |  | | ------ | 0.249***** |
| No | 100 (83.3) | 28 (93.3) | |  |  |
| Yes | 20 (16.7) | 2 (6.7) | |  |  |
| **antihypertensive medication** |  |  | | 0.002 | 0.967 |
| No | 71 (59.2) | 17 (56.7) | |  |  |
| Yes | 49 (40.8) | 13 (43.3) | |  |  |
| **antidiabetic medication** |  |  | | ------ | 0.786***** |
| No | 100 (83.3) | 26 (86.7) | |  |  |
| Yes | 20 (16.7) | 4 (13.3) | |  |  |
| **pain reliever** |  |  | | ------ | 0.583***** |
| No | 115 (95.8) | 30 (100.0) | |  |  |
| Yes | 5 (4.2) | 0 (0.0) | |  |  |
| **sleeping aid** |  |  | | ------ | 0.186* |
| No | 116 (96.7) | 28 (93.3) | |  |  |
| Yes | 4 (3.3) | 2 (6.7) | |  |  |
| **cough suppressant** |  |  | | ------ | > 0.999* |
| No | 117 (97.5) | 30 (100.0) | |  |  |
| Yes | 3 (2.5) | 0 (0.0) | |  |  |
| **hormonal medication** |  |  | | ------ | > 0.999* |
| No | 118 (98.3) | 30 (100.0) | |  |  |
| Yes | 2 (1.7) | 0 (0.0) | |  |  |
| **thrombolytic agent** |  |  | | ------ | > 0.999* |
| No | 117 (97.5) | 29 (96.7) | |  |  |
| Yes | 3 (2.5) | 1 (3.3) | |  |  |
| **aspirin** |  |  | | ------ | 0.688* |
| No | 112 (93.3) | 29 (96.7) | |  |  |
| Yes | 8 (6.7) | 1 (3.3) | |  |  |
| ***Dietary intake levels*** | | | | | |
| **rice** |  |  | | ------ | 0.083***** |
| ≤ 100 | 46 (38.3) | 6 (20.0) | |  |  |
| 100~ | 26 (21.7) | 12 (40.0) | |  |  |
| 210~ | 41 (100.0) | 9 (30.0) | |  |  |
| > 450 | 7 (5.8) | 3 (10.0) | |  |  |
| **flour** |  |  | | ------ | 0.579***** |
| ≤ 21.50 | 25 (20.8) | 3 (10.0) | |  |  |
| 21.50~ | 18 (15.0) | 5 (16.7) | |  |  |
| 50~ | 35 (29.2) | 9 (30.0) | |  |  |
| > 100 | 42 (35.0) | 13 (43.3) | |  |  |
| **coarse grain** |  |  | | **------** | **0.001*** |
| ≤ 40 | 32 (26.7) | 18 (60.0) | |  |  |
| 40~ | 29 (24.2) | 5 (13.3) | |  |  |
| 100~ | 36 (30.0) | 4 (16.7) | |  |  |
| > 143 | 23 (19.2) | 3 (10.0) | |  |  |
| **root and tuber vegetables** |  |  | | ------ | 0.104***** |
| ≤ 36 | 52 (43.3) | 10 (33.3) | |  |  |
| 36~ | 35 (29.2) | 15 (50.0) | |  |  |
| 100~ | 15 (12.5) | 4 (13.3) | |  |  |
| > 170 | 18 (15.0) | 1 (3.3) | |  |  |
| **vegetables** |  |  | | ------ | 0.137***** |
| ≤ 25 | 42 (35.0) | 17 (56.7) | |  |  |
| 150~ | 34 (28.3) | 8 (26.7) | |  |  |
| 200~ | 22 (18.3) | 2 (6.7) | |  |  |
| > 300 | 22 (18.3) | 3 (1.0) | |  |  |
| > 90 | 26 (21.7) | 9 (30.0) | |  |  |
| **beef** |  |  | | 5.55 | 0.135 |
| ≤ 2.7 | 31 (25.8) | 4 (13.3) | |  |  |
| 2.7~ | 28 (23.3) | 7 (23.3) | |  |  |
| 7.14~ | 36 (30.0) | 7 (23.3) | |  |  |
| > 14.3 | 25 (20.8) | 12 (4.0) | |  |  |
| **freshwater fish** |  |  | | 3.24 | 0.356 |
| ≤ 14 | 27 (22.5) | 6 (20.0) | |  |  |
| 14~ | 34 (28.3) | 13 (43.3) | |  |  |
| 34~ | 30 (25.0) | 4 (13.3) | |  |  |
| > 65 | 29 (24.2) | 7 (23.3) | |  |  |
| **animal offal** |  |  | | **------** | **< 0.001*** |
| ≤ 0.41 | 102 (85.0) | 14 (47.0) | |  |  |
| 0.41~ | 6 (5.0) | 0 (0.0) | |  |  |
| 1.64~ | 7 (5.8) | 1 (3.3) | |  |  |
| > 4.30 | 5 (4.2) | 1550.0) | |  |  |
| **yogurt** |  |  | | ------ | 0.292***** |
| ≤ 35.7 | 94 (78.3) | 28 (93.3) | |  |  |
| 35.7~ | 10 (8.3) | 0 (0.0) | |  |  |
| 53.6~ | 7 (5.8) | 1 (3.3) | |  |  |
| > 125 | 10 (8.3) | 1 (3.3) | |  |  |
| **egg** |  |  | | 0.018 | 0.893 |
| ≤ 50 | 84 (70.0) | 22 (73.3) | |  |  |
| > 50 | 36 (30.0) | 8 (26.7) | |  |  |
| **milk** |  |  | | 1.62 | 0.203 |
| ≤ 66 | 68 (56.7) | 12 (40.0) | |  |  |
| > 66 | 52 (43.3) | 17 (56.7) | |  |  |
| **plant oil** |  |  | | ------ | 0.181***** |
| ≤ 15 | 31 (25.8) | 8 (26.7) | |  |  |
| 15~ | 34 (28.3) | 5 (16.7) | |  |  |
| 30~ | 52 (43.3) | 17 (56.7) | |  |  |
| > 60 | 19 (15.8) | 1 (3.3) | |  |  |
| **poultry** |  |  | | 0.042 | 0.838 |
| ≤ 66 | 59 (49.2) | 16 (53.3) | |  |  |
| > 66 | 61 (50.8) | 14 (46.7) | |  |  |
| **mutton** |  |  | | 294 | 0.588***** |
| ≤ 2.77 | 92 (76.7) | 25 (83.3) | |  |  |
| > 2.77 | 28 (23.3) | 5 (16.7) | |  |  |
| **seafood** |  |  | | 5.074 | 0.879 |
| ≤ 4.90 | 85 (70.8) | 21 (70.0) | |  |  |
| > 4.90 | 35 (29.2) | 9 (30.0) | |  |  |
| **bean products** |  |  | | 0.082 | 0.774 |
| ≤ 4.60 | 62 (51.7) | 17 (23.3) | |  |  |
| > 4.60 | 58 (24.3) | 13 (76.7) | |  |  |
| **fruit** |  |  | | 3.192 | 0.070 |
| ≤ 150 | 44 (36.7) | 17 (70.0) | |  |  |
| > 150 | 76 (63.3) | 13 (30.0) | |  |  |
| **pickles** |  |  | | 0.355 | 0.552***** |
| ≤ 10.43 | 89 (74.2) | 20 (66.7) | |  |  |
| > 10.43 | 31 (25.8) | 10 (33.3) | |  |  |
| **desserts** |  |  | | 0.366 | 0.545 |
| ≤ 20.67 | 87 (72.5) | 24 (80.0) | |  |  |
| > 20.67 | 33 (27.5) | 6 (20.0) | |  |  |
| **dried Fruits** |  |  | | 0.002 | 0.964 |
| ≤ 25.71 | 85 (70.8) | 22 (73.3) | |  |  |
| > 25.71 | 35 (29.2) | 8 (26.7) | |  |  |
| **animal Fat** |  |  | | 2.321 | 0.128 |
| ≤ 45.6 | 107 (89.2) | 30 (100.0) | |  |  |
| > 45.6 | 13 (10.8) | 0 (0) | |  |  |

A total of 62 factors in 5 main categories (demographic characteristics, disease histories, lifestyle habits, medical contact histories and dietary intake levels) were used for univariate analysis. Daily intake of foods that conform to a symmetrical distribution was categorized into quartiles, while those Not adhering to a symmetrical distribution were grouped using the median. *P** calculated by Fisher's exact test.
